# Supplementary material for: Heat adaptation of phage T7 under an extended genetic code
Source: Virus Evol. 2021 Dec 1;7(2):veab100. doi: 10.1093/ve/veab100 (PMC8923235; doi:10.1093/ve/veab100)
Supplement: veab100_Supp [file veab100_supp.zip › Sequencing_Analysis.zip!/Sequencing_Analysis/A1/evidence/JC_SIDE_1_66.html]

BRESEQ :: Evidence


| New junction evidence | | | | | | | | | | | |
| --- | --- | --- | --- | --- | --- | --- | --- | --- | --- | --- | --- |
|  | | seq id | position | reads (cov) | reads (cov) | score | skew | freq | annotation | gene | product |
| \* | ? | NC\_001604.1 | = 36882 | 3450 (1.120) | 16 (0.010) | 15/582 | 37.4 | 0.48% | intergenic (–/–) | *–/–* | –/– |
|  | ? | NC\_001604.1 | 37265 = | 3289 (1.100) |  |  |  |  | intergenic (–/–) | *–/–* | –/– |
| Rejected: Coverage evenness skew score above cutoff. | | | | | | | | | | | |
| Rejected: Frequency below/above cutoff threshold. | | | | | | | | | | | |

Only 100 of 3464 total aligned reads displayed.  

`CTTAGAGATGTTGGACACTGCGATGGCTCAGCGTATGCTTGCGGACCTTTCGGACCATGAGCGTCGCTCTCCGCAACTCTATAATGCTATTAACAAACTGTTAGACCGCCACAAGTTCCAGATTGGTAAGTTGCAGCCGGATGTTCACATCTTAGGTGGCCTTGCTGGTGCTCTTGAAGAGTACAAAGAGAAAGTCGGTGATAACGGTCTTACGGATGATGATATTTACACATTACAGTGATATACTCAAGGCCACTACAGATAGTGGTCTTTATGGATGTCAT‑TGTCTATACGAGATGCTCCTACGTGAAATCTGAAAGTTAACGGGAGGCATTATGCTAGAATTTTTACGTAAGCTAATCCCTTGGGTTCTCGCTGGGATGCTATTCGGGTTAGGATGGCATCTAGGGTCAGACTCAATGGACGCTAAATGGAAACAGGAGGTACACAATGAGTACGTTAAGAGAGTTGAGGCTGCGAAGAGCACTCAAAGAGCAATCGATGCGGTATCTGCTAAGTATCAAGAAGACCTTGCCGCGCTGGAAGGGAGCACTGATAGGATTATTTCTGATTTGCGTAGCGACAATAAGCGGTTGCGCGTC  >  NC_001604.1/36582‑37183`  

                                                                                                                                                                                                                                                                                                             `|`                                                                                                                                                                                                                                                                                                               
  `gacagaTGTTGGACACTGCGATGGCTCAGCTTATGCTGGCGGACCTTGCGGACCATGAGCGTCGCTCTCCGCAACTCTATAATGCTATTAACAAACTGTTAGACCGCCACAAGTTCCAGATTGGTAAGTTGCAGCCGGATGTGCACATCTTAGGTGGCCTTGCTGGTGCTCTTGAAGAGTACAAAGAGAAAGTCGGTGATAACGGTCTTACGGATGATGATATTTACACATTACAGTGATATACTCAAGGCCACTACAGATAGTGGTCTTTATGGATGTCAT‑TGTCTATACGAGATGCTcc                                                                                                                                                                                                                                                                                                             <  1:176679/298‑1 (MQ=255)`  
         `gatGGACACTGCGATGGCTCAGCGTATGCTTGCGGACCGTTCAGACCATGAGAGTCGGTCTCCGCAACTCGATAATGTTATTAACAAACTGTTAGACCGCCACAAGTTCAAGATTGGTAAGTTGCAGCCGGATGTTCACATCTTAGGTGGCCTTGCTGGTGCTATTGAAGAGTACAAAGAGAAAGTCGGTGAGAACGGTCTTACGGATGATGATATTTACACAATACAGTGATATACTCAAGGCCACTACAGATAGTGGTCTTTATGGATGTCAT‑TGTCTATACGAGATGCTCCTACGTGa                                                                                                                                                                                                                                                                                                      <  1:453066/299‑1 (MQ=255)`  
         `gTTGGACACTGCGATGGCTCAGCGTATGCTTGCGGACCTTTCGGACCATGAGCGTCGCTCTCCGCAACTCTATAATGCTATTAACAAACTGTTAGACCGCCACAAGTTCCAGATTTGTAAGTTGCAGCCGGATGTTCACATCTTAGGTGGCCTTGCTGGTGCTTTTGAAGAGTACAAAGAGAAAGTCGGTGATAACGGTCTTACGGATGATGATATTTACACATTACAGTGATATACTCAAGGCCACTACAGATAGTGGTCTTTATGGATGTCAT‑TGTCTATACGAGATGCTCCTACGTGa                                                                                                                                                                                                                                                                                                      <  1:234846/301‑1 (MQ=255)`  
                 `cTGCGATGGCTCAGCGTATGCTTGCGGACCTTTCGGACCATGAGCGTCGCTCTCCGCAACTCTATAATGCTATTAACAAACTGTTAGACCGCCACCAGTTCCAGATTGGTCAGTTGCAGCCGGATGTTCACATCTTAGGTGGCCTTGCTGGTGCTCTTGAAGAGTACACAGAGAAAGTCGGTGATAACGGTCTTACGGATGATGATATTTCCACATTACAGTGATATACTCAAGGCCACTACAGATAGTGGTCTTTATGGCTGTCAT‑TGTCTATACGAGATGCTccctgtctcttatacac                                                                                                                                                                                                                                                                                              >  1:56831/1‑286 (MQ=255)`  
                 `cTGCGATGGCTCAGCGTATGCTTGCGGACCTTTCGGACCATGAGCGTCGCTCTCCGCAACTCTATAATGCTATTAACAAACTGTTAGACCGCCACAAGTTCCAGATTGGTAAGTTGCAGCCGGATGTTCACATCTTAGGTGGCCTTGCTGGTGCTCTTGAAGAGTACAAAGAGAAAGTCGGTGATAACGGTCTTACGGATGATGATATTTACACATTACAGTGATATACTCAAGGCCACTACAGATAGTGGTCTTTATGGATGTCAT‑TGTCTATACGAGATGCTCCTACGTGAAATCTGaa                                                                                                                                                                                                                                                                                              >  1:100348/1‑301 (MQ=255)`  
                        `ggCTCAGCGTATGCTTGCGGACCTTTCGGACAATGAGCGTCGCTCTCCGCAACTCTATAATGCTATTAACAAACTGTTAGACCGCCACAAGTTCCAGATTGGTAAGTTGCAGCCGGATGTTCACATCTTAGGTGGCCTTGCTGGTGCTCTTGAAGAGTACAAAGAGAAAGTCGGTGATAACGGTCTTACGGATGATGATATTTACACATTACAGTGATATACTCAAGGCCACTACAGATAGTGGTCTTTATGGATGTCAT‑TGTCTATACGAGATGCTCCTACGTGAAATCTGAAAGTTAAc                                                                                                                                                                                                                                                                                       >  1:507718/1‑301 (MQ=255)`  
                        `agacaggCGTATGCTTGCGGACCTTTCTGACCATGCGCGTCGCTCTCCGCAACTCTATAATGCTATTAACAACCTTTTAGACCGCCACAAGTTCCAGATTGGTAAGTTGCAGCCGGATTTTCACATCTTAGGTGGCCTTGCTGGTGCTCTTGAAGAGTACAAAGAGAAAGTCGGTGATAACTGTCTTACGGATGATGATATTTACACATTACAGTGATATACTCAAGGCCACTACAGATAGTGGTCTTTATGGATGTAAT‑TGTCTATACAAGATGCTCCTACGTgctgtctcttatacacc                                                                                                                                                                                                                                                                                       >  1:195312/7‑285 (MQ=255)`  
                             `aGCGTATGCTTGCGGACCTTTCGGACCATGAGCGTCTCTCTCCGCAACTCTATAATGCTATTAACAAACTGTTAGACCGCCACAAGTTCCAGATTGGTAAGTTGCAGCCGGATGTTCACATCTTAGGTTGCCTTGCTGGTGCTCTTGAAGAGTACAAAGAGAAAGTCGGTGATAACGGTCTTACGGATGATGATATTTACACATTACAGTGATATACTCAAGGCCACTACAGATAGTGGTCTTTATGGATGTCAT‑TGTCTATACGAGATGTTCCTACGTGAAATCTGAAAGTTAACTGGAg                                                                                                                                                                                                                                                                                  >  1:414542/1‑301 (MQ=255)`  
                                `gTATGCTTGCGGACCTTTCGGACCATGAGCGTCGCTCTCCGCAACTCTATAATGCTATTAACAAACTGTTAGACCGCCACAAGTTCCATATTGGTAAGTTGCAGCCGGATGTTCACATCTTAGGTGGCCTTGCTGGTGCTCTTGAAGAGTACAAAGAGAAAGTCGGTGATAACGGTCTTACGGATGATGCTATTTACACATTACAGTGATATACTCAAGGCCACTACAGCACGCGGTCTTTATGGATGTCAT‑TGTCTATACGAGATGCTACTACGCTAACTCTGAAAGTTAACGGGAGGCa                                                                                                                                                                                                                                                                               >  1:91012/1‑301 (MQ=255)`  
                                                   `ggACCATGAGCGCAGCTCGCCTCAACTCTATAATGCTATTAACAAACTGTTATACCGCCACAAGTTCCAGATTGGTAAGTTGCAGCCGGATGTTCACATCTTAGGTGGCCTTGCTGGTGCTCTTGAAGAGTACAAAGAGAAAGTCGGTGATAACGGTCTTACGGATGATGATATTTACACATTACAGTGATATACTCAAGGCCACTACAGATAGTGGTCTTGATGGATGTCAT‑TGTCTATACGAGATGCTCCTACGTGAAATCTGAAAGTTAACGGGAGGCATTATGCTAGAATTTTTACg                                                                                                                                                                                                                                                            <  1:95406/301‑1 (MQ=255)`  
                                                              `agagacagccGCAACTCTATAATGCTATTAACACACTGTTAGACCGCCACAAGTTCCAGATTGGTAAGTTGCAGCCGAATTTTCACATCTTAGGTGGCCTTGCTGGTGCTATTGAAGAGTACAAAGAGAAAGTCGGTTATAACGGTCTTACGGATGATGATATTTACACATTACAGTGATATACTCAAGGCCACTACAGATAGTGGTCTTTATGGATGTCAT‑TGTCTATACGAGATGCTCCTACGTGAAATCTGAAAGTTAACGGGAGGCATTATGCTAGAATTTTTACGTAAGCTAATcc                                                                                                                                                                                                                                                 <  1:225574/293‑1 (MQ=255)`  
                                                                 `gCTCTCCGCAACTCTATAATGCTATTAACAAACTATTAGACCGCCACAAGTTCCAGATTGGTAAGTTGCAGCCGGATGTTCACATCTTAGGTGGCCTTGCTTGTGCTCTTGAAGAGTACAAAGAGAAAGTCGGTGATAACGGTCTTACGGATGATGATATTTCAACATTACAGTGAGTTACTCAAGGCCACTACAGATAGTGGTCTTTATGGATGTCAT‑TGTCTATACGAGATGCTCCTACGTGAAGTCAGAAAGTTAACGGGAGGCATTATGATAGAATTTTTACGTAAGCTAATCCCtt                                                                                                                                                                                                                                              >  1:251981/1‑301 (MQ=255)`  
                                                                    `ctcCGCAACTCTATAATGCTATTAACAAACTGTTAGACCGCCACAAGTTCCAGATTGGTAAGTTGCAGACGGATGTTCACATCTTAGGTGGCCTTGCTGGTGCTCTTGAAGAGTACAAAGAGAAAGTCGGTGATAACGGTCTTACGGATGATGATATTTACACATTACAGTGATATACTCAAGGCCACTACAGATAGTGGTCTTTATGGATGTCAT‑TGTCTATACGAGATGCTACTACGTGAAATCTGAAAGTTAACGGGAGGAATTATGCTAGAATTTTTACGTAAGCTAATCCCTTggg                                                                                                                                                                                                                                           <  1:403507/301‑1 (MQ=255)`  
                                                                     `tcCACAACACTATAATGCTATTAACAAACTGAGAGACCGACACAAGTTCCAGATTGGTAAGGTGCAGCCGGATGTACACATCTAAGGTGGACTTGGTGGTGATCTTGAGGAGTACAAAGGGAAAGTCGATGATGACAGTCTTTCGGATGATGATATTTACACAATACAGTGATATACTCAAGGCCACTACAGATAGTGGTCATTATGGATGTAAT‑TGTCTCTACGAGATGCTCCTACGTGAAATCTGCAAGTTGACGGGAGTCATTATGCTAAAATTTTTACGTAAGCTAATCCCTTGGGt                                                                                                                                                                                                                                          <  1:431176/301‑1 (MQ=255)`  
                                                                      `ccGCAACTCTATAATGCTATTAACAAACTGTTAGACCGCCACAAGTTACAGATTGGTAAGTTGCAGCCGGATGTTCACATCTTAGGTGGCCTTGCTGGTGCTCTTGAAGAGTACAAAGAGAAAGTCGGTGATAACGGTCTTACGGATGATGTTATTTACACATTACAGTGATATACTCAAGGCCACTACAGATAGTGGTCTTTATGGATGTCAT‑TGTCTATACGAGATGCTCCTACGTGAAATCTGAAAGTTAACCGGAGGCATTATGCTAGAATTTTTACGTAAGCTAATTCCtgtctct                                                                                                                                                                                                                                         >  1:462339/1‑295 (MQ=255)`  
                                                                           `aCTCTATAATGCTATTAACAAACTGTTAGACCGCCACAAGTTCCAGATTGGTAAGTTGCAGCTGGATGTTCACATCTTAGGTGGCCTTGCTGGTGCTCTTGAAGAGTACAAAGAGAAAGTCGGTGATAACGGTCTTACGGATGATGATATTTACACATTACAGTGATATACTCAAGGCCACTACAGATAGTGGTCTTTATGGATGTCAT‑TGTCTATACGAGATGCTCCTACGTGAAATCTGAAAGTTAACGGGAGGCATTATGCTAGAATTTTTACGTAAGCTAATCCCTTGGGTTCTCGc                                                                                                                                                                                                                                    >  1:374818/1‑301 (MQ=255)`  
                                                                           `aCTCTATAATGCTATTAACAAACTGTTAGACCGCCACAAGTTCCAGATTGGTAAGTTGCAGCCGGATGTTCACATCTTAGGTGGCCTTGCTGGTGCTCTTGAAGAGTACAAAGAGAAAGTCGGTGATAACGGTCTTACGGATGATGATATTTACACATTACAGTGATATACTCAAGGCCACTACAGATAGTGGTCTTTATGGATGTCAT‑TGTCTATACGAGATGCTCCTACGTGAAATCTGAAAGTTAACGGGAGGCATTATGCTAGAATTTTTACGTAAGCTAATCCCtgtctcttatac                                                                                                                                                                                                                                    >  1:409012/1‑290 (MQ=255)`  
                                                                            `ctctATAATGCTATTAACAAACTGTTAGACCTCCACAAGTTCCAGATTGGTAAGTTGCAGCCGGATGTTCACATCTTAGGTGGCCTTGCTGGTGCTCTTGAAGAGTACAAAGAGAAAGTCGGTGATAACGGTCTTACGGATGATGATATTTACACATTACAGTGATATACTCAAGGCCACTACAGATAGTGGTCTTTATGGATGTCAT‑TGTCTATACGAGATGCTCCTACGTGAAATCTGAAAGTTAACGGTAGGCATTATGCTAGAATTTTTACGTAAGCTAATCCCTTGGGTTCTCGcc                                                                                                                                                                                                                                   >  1:36027/1‑300 (MQ=255)`  
                                                                            `ctctATAATGCTATTAACAAACTGTTAGACCGCCACAAGTTCCAGATTGGTAAGTTGCAGCCGGATGTTCACATCTTAGGTGGCCTTGCTGGTGCTCTTGAAGAGTACAAAGAGAAAGTCGGTGATAATGGTCTTACAGATGATGATATTTCCACATTACAGTGATATACTCAAGGCCACTACAGATAGTGGTCTTTATGGATGTCAT‑TGTCTATACGAGATGCTCCTACGTGAAATCTGAAAGTTAACGGGAGGCATTATGCTAGAATTTTTACGTAAGCTAATCCCTTGGGTTCTCGCt                                                                                                                                                                                                                                   >  1:239460/1‑301 (MQ=255)`  
                                                                             `tctATAATGCTATTAACAAACTGTTAGACCGCCACAAGTTCCAGATTGGTAAGTTGCAGCCGAATGTTCACATCTTAGGTGGCCTTGCTGGTGCTCTTGAAGAGTACAAAGAGAAAGTCGGTGATAACGGTCTTACGGATGCTGATATTTACACATTACAGTGATATACTCAAGGCCACTACAGATAGTGGTCTTTATGGATGTCAT‑TGTCTATACGAGATGCTCCTACGTGAAATCTGCAAGTTAACGGGAGGCATTATGCTAGAATTTTTACGTAAGCTAATCCCTTGGGTTCTCGCTg                                                                                                                                                                                                                                  >  1:314610/1‑301 (MQ=255)`  
                                                                              `ctATAATGCTATTAACAAACTGTTAGACCGCCACAAGTTCCAGATTGGTAAGTTGCAGCCGGATGTTCACATCTTAGGTGGCCTTGCTGGTGCTCTTGAAGAGTACAAAGAGAAAGTCGTTGATAACGGTCTTACGGATGATGATATTTACACATTACAGTGTTATACTCAAGGCCACTACAGCTAGTGGTCTTTATGGATGTCAT‑TGTCTATACGAGATTCTCCTACGTGAAATCTGAAAGTTAACGGGAGGCATTATGCTAGAATTTTTACTTCAGCTAATCCCTTGGGTTCTCGCTgg                                                                                                                                                                                                                                 >  1:371213/1‑301 (MQ=255)`  
                                                                              `ctATAATGCTATTAACAAACTGTTAGACCGCCACAAGTTCCAGATTGGTAAGTTGCAGCCGGATGTTCACATCTTAGGTGGCCTTGCTGGTGCTCTTGAAGAGTACAAAGAGAAAGTCGGTGATAACGGTCTTACGGATGATGATATTTACACATTACAGTGATATACTCAAGGCCACTACAGATAGTGGTCTTTATGGATGTCAT‑TGTCTATACGAGATGCTCCTACGTGAAATCTGAAAGTTAACGGGAGGCATTATGCTAGCATTTTTACGTAAGATAATCCCtgtctcttatacaca                                                                                                                                                                                                                                 >  1:71334/1‑287 (MQ=255)`  
                                                                              `ctATAATGCTATTAACAAACTGTTAGACCGCCACAAGTTCCAGATTGGTAAGTTGCAGCCGGATGTTCACATCTTAGGTGGCCTTGCTGGTGCTCTTGAAGAGTACAAAGAGAAAGTCGGTGATAACGGTCTTACGGATGATGATATTTACACATTACAGTGATATACTCAAGGCCACTACAGATAGTGGTCTTTATGGATGTCAT‑TGTCTATACGAGATGCTCCTACGTGAAATCTGAAAGTTAACGGGAGGCATTATGCTAGAATTTTTACGTAAGCTAATCCCTTTGGTTCTCGcctg                                                                                                                                                                                                                                 >  1:503200/1‑298 (MQ=255)`  
                                                                              `ctATAATGCTATTAACAAACTGTTAGACCGCCACAAGTTCCAGATTGGTAAGTTGCAGCCGGATGTTCACATCTTAGGTGGCCTTGCTGGTGCTCTTGAAGAGTACAAAGAGAAAGTCGGTGATAACGGTCTTACGGATGATGATATTTACACATTACAGTGATATACTCAAGGCCACTACAGATAGTGGTCTTTATGGATGTCAT‑TGTCTATACGAGATGCTCCTACGTGAAATCTGAAAGTTAACGGGAGGCATTATGCTAGAATTTTTACGTAAGCTAATCCCTTGGGTTCTCGCTgg                                                                                                                                                                                                                                 >  1:267541/1‑301 (MQ=255)`  
                                                                                `agatgtgtataagagacaggTTAGACCGCCACAAGTTCCAGATTGGTAAGTTGCAGCCGGATGTTCACATCTTAGGTGGCCTTGCTGGTGCTCTTGAAGAGTACAAAGAGAAAGTCGGTGATAACGGTCTTACGGATGATGATATTTACACATTACAGTGATATACTCAAGGCCACTACAGATAGTGGTCTTTATGGATGTCAT‑TGTCTATACGAGATGCTCCTACGTGAAATCTGAAAGTTAACGGGAGGCAGTATGCTAGAATTTTTACGTAAGCTAATCCCTTGGGTTCTCGCTGGGa                                                                                                                                                                                                                               <  1:447203/282‑1 (MQ=255)`  
                                                                                        `aatAACAAACTGTTAGACCGACACAGGTTCCAGAATGGTAAGTTGCAGCCGGAGGTTGAGATCTTAGGTGGCCTTGCTGGTGCGCTTGAAGAGTGCAAAGAGAAAGTCGGTGAGAACGGTCTTACGGAGGATGATATTTACACATTACAGTGATATACTCAAGGCCAATACAGTTAGTGGTCTTTATGGATGTCAT‑TGTCTATACGAGATGCTCCTACGTGAAATCTGAAAGTTAACGGGAGGCATTATGCTAGAATTTTTACGTAAGCTAATCCCTTGGGTTCTCGGTGGGATGCTAGTc                                                                                                                                                                                                                       <  1:439280/299‑1 (MQ=255)`  
                                                                                          `tAACAAACTGTTAGACCGCCACAAGTTCCAGATTGGTAAGTTGCGGCCGGATGTTCACATCTTAGGTGGCCTTGCTGGTGCTCTTGAAGAGTACAAAGAGAAAGTCGGTGATAACGGTCTTACGGATGATGATATTTACACATTACAGTGATATACTCAAGGCCACTACAGATAGTGGTCTTTATGGATGTCAT‑TGTCTATACGAGATGCTCCTACGTGAAATCTGAAAGTTAACGGGAGGCATTATGCTAGAATTTTTACGTAAGCTAATCCATTGGGTTCTCGCTGGGATGCTATTCgg                                                                                                                                                                                                                     >  1:75851/1‑301 (MQ=255)`  
                                                                                                 `cTGTTAGACCGCCACAAGTTCCAGATTGGTAAGTTGCAGCCGGATGTTCACATCTTAGGTGGCCTTGCTGGTGCTCTTGAAGAGTACCAAGAGAAAGTCGGTGATAACGGTCTTACGGATGATGATGTTTACACATTATAGTGATATACTAAAGGCCACTACATATAGTGGTCTTTATGGATGTCAT‑TGTCTATACGAGATGCTCCTACGTGAAATCTGAAAGTTAACGGGATGCATTATGCTAGAATTTTTACGTAAGCTAATCCCTTgggctatctcttatacacatctccgagcccac                                                                                                                                                                                                              >  1:109203/1‑272 (MQ=255)`  
                                                                                                 `cTGTTAGACCGCCACAAGTTCCAGATTGGTAAGTTGCAGCCGGATGTTCACATCTTAGGTGGCCTTGCTGGTGCTCTTGAAGAGTACAAAGAGAAAGTCGGTGATAACGGTCTTACGGATGATGATATTTACACATTACAGTGATATACTCAAGGCCACTACAGATAGTGGTCTTTATGGATGTCAT‑TGTCTATACGAGATGCTCCTACGTGAAATCTGAAAGTTAACGGGAGGCATTATGCTAGAATTTTTACGTAAGCTAATCCCTTGGGTTCTCGcctgtctcttatacacatctccg                                                                                                                                                                                                              >  1:185354/1‑279 (MQ=255)`  
                                                                                                 `cTGTTAGACCGACACAAGTTCCAGATTGGTAAGTTGCAGCCGGATGTTCACATCTTAGGTGGCCTTGCTGGTGCTCTTGAAGAGTACAAAGAGAAAGTCGGTGATAACGGTCTTACGGATGATGATATTTACACATTACAGTGATATACTCAAGGCCACTACAGATAGTGGTCTTTATGGATGTCAT‑TGTCTATACGAGATGCTCCTACGTGAAATCTGAAAGTTAACGGGAGGCATTATGCTAGAATTTTTACGTAAGCTAATCCCTTGGGTTCTCGCTGGGATGCTATTAGGGTTAGGa                                                                                                                                                                                                              >  1:332659/1‑301 (MQ=255)`  
                                                                                                  `tGTTAGACTTCCACACGTTCCAGATTGGTAAGTTGCAGCCGGATGTTCAAATCTTAGGTGGTCTTGCTGGTGCTCTTGAAGAGTACAAAGAGAAAGTCGGTGATAGCGGTCTTACGGATGATGATATTTACACATTACAGTGATATACTCAAGGCCACTACAGATAGTGGTCTTTATGGATGTCAT‑TGTCTATACGAGATGCTCCTACGTGAAATCTGAAAGTTAACGGGAGGCATTATGCTAGAATTTTTACGTAAGCTAATCCCTTGGGTTCTCGCTGGGATGCTATTCGGGTTAGGAt                                                                                                                                                                                                             <  1:259110/301‑1 (MQ=255)`  
                                                                                                                   `ttCCAGATTGGTAAGTTGCAGCCGGATGTTCACATCTTAGGTGGCCTTGCTGGTGCTCTTGAAGAGTACAAAGAGAAAGTCAGTGGTAACGGTCTTACGGATGATGATATTTACACATTACAGTGATATACTCAAGGCCACTACAGATAGTGGTCTTTATGGATGTCAT‑TGTCTATACGAGATGCTCCTACGTGAAATCTGAAAGTTAACGGGAGGCATTATGCTAGAATTTTTACGTAAGCTAATCCCTTGGGTTCTCGCTGGGATGCTATTCGGGTTAGGATGGCATCTAGGGTCAGAc                                                                                                                                                                                            <  1:199102/301‑1 (MQ=255)`  
                                                                                                                     `ccAGATTGGGAAGTTGCAGCCGGATGTTCACATCTTAGGTGGACTTTCTGGTGCTCTTGAAGAGTACAAAGAGAAAGTCGGTGATAACGGTCTTACGGATGATGATATTTACACATTACAGTGATATACTCAAGGCCACTACAGATAGTGGTCTTTATGGATGTCAT‑TGTCTATACGAGATACTCCTACGTGAAATCTGAAAGTTAACGGGAGGCATTATGCTAGAATTTTTACGTAAGCTAATCCCTTGGGTTCTCGCTGGGATGCTATTCGGGTTATGATGGCATCTAGGGTCAGACTc                                                                                                                                                                                          <  1:422394/301‑1 (MQ=255)`  
                                                                                                                                   `tccAGCCGGATGTTCACATCTTAGGTGGCCTTGCTGGTGCTCGTGAAGAGTACAAAGAGAAAGTCGGTGATAACGGTCTTACGGATGATGATATTTACACATTACAGTGATATACTCAAGGCCACTACAGATAGTGGTCTTTATGGATGTCAT‑TGTCTATACGAGATGCTCCTACGTGAAATCTGAAAGTTAACGGGAGGCATTATGCTAGAATTTTTACGTAAGCTAATCCCTTGGGTTCTCGCTGGGATACTATTCTGGTTAGGATGGCATCTAGGGTCAGACTCAATGGACGCTAAAt                                                                                                                                                                            <  1:211421/299‑1 (MQ=255)`  
                                                                                                                                                    `aTCTTAGGTGGCCTTGCTGGTGCCCTTGAAGAGTACAAAGAGAAAGTCGGTGATAACGGTCTTACGAATGATGATATTTACACATTACAGTGATATACTCAAGGCCACTACAGATAGTGGTCTTTATGGATGTCAT‑TGTCTATACGAGATGCTCCTACGTGAAATCTGAAAGTTAACGGGAGGCATTATGCTAGAATTTTTACGTAAGCTAATCCCTTGGGTTCTCGCTGGGATGCTATTCGGGTTAGGATGGCATCTAGGGTCAGACTCAATGGACGCTAAATGGAAACAGGAGGTacac                                                                                                                                                           <  1:11270/301‑1 (MQ=255)`  
                                                                                                                                                        `tAGGTGGCCTTGCTGGTGCTCTTGAAGAGTACAAAGAGAAAGTCGGTGATAACGGTCTTACGGATGATGATATTTACACATTACAGTGATATACTCAAGGCCACTACAGATAGTGGTCTTTATGGATGTCAT‑TGTCTATACGAGATGCTCCTACGTGAAATCTGAAAGTTAACGGGAGGCATTATGCTAGAATTTTTACGTAAGCTAATCCCTTGGGTTATCGCTGGGATGCTATTCGGGTTAGGATGGCATCTAGGGTCAGACTCAATGGATGCTAAATGGAAACAGGAGGTacaatgag                                                                                                                                                       >  1:324647/1‑296 (MQ=255)`  
                                                                                                                                                        `tAGGTGGCCTTGCTGGTGCTCTTGAAGAGTACAAAGAGAAAGTCGGTGATAACGGTATTACGGATGATGATATTTACCCATTAAAGTGATATACTCAAGGCCACTACAGATAGTTGTCTTTATGGATGTCAT‑TGTCTATACGAGATGCTCCTCTGTGAAATCTGAACGTTAACGGGAGGCATTATGCTAGACTTTTTCCGTAAGCTAATCCCTTGGGTTCTCGCTGGGATGCTATTCGTGTTAGGCTGGTATCTAGGGTCAGACTCCACGGACGCTAAATGGAAACAGGAGGTacaatgag                                                                                                                                                       >  1:248232/1‑296 (MQ=255)`  
                                                                                                                                                           `gTGGCCTTGCTGGTGCTCTTGAAGAGTACAAAGAGAAAGTCGGTGATAACGGTCTTACGGATGATGATATTTACACATTACAGTGATATACTCAAGGCCACTACAGATAGTGGTCTTTATGGATGTCAT‑TGTCTATACGAGATGCTCCTACGTGAAATCTGAAAGTTAAAGGGAGGCATTATGCTAGAATTTTTACGTAAGCTAATCCCTTGGGTTCTCGCTGGGATGCTATTCGGGTTAGGATGGCATCTAGGGTCAGACTCAATGGACGCTAAATGGAAACAGGAGGT‑‑ACAATGAGTAc                                                                                                                                                  <  1:120679/301‑1 (MQ=255)`  
                                                                                                                                                                 `tggCTGGGGCGCTTGAAGAGGACAAAGAGAAAGTCGGTGATAACGGTCTTACGGAGGATGATATTTACACATTACAGTGATATACTCAAGGCCGCTACAGATAGTGGGCGTTATGGATGTCAT‑TGTCTATACGAGATGCTCCTACGTGAAATCTGAAAGTTAACGGGAGGCATTATGCTAGAATGTTTACGTAAGCTAATCCCTTGGGTTCTCGCTGGGATGCTATTCGGGTTAGGATGGCATCTAGGGTCAGACTCAATGGACGCTAAATGGAAACAGGAGGT‑‑ACAATGAGTACGTTAag                                                                                                                                            <  1:385880/299‑1 (MQ=255)`  
                                                                                                                                                                  `gctcggagatgggtaaaagagacaggagaAAGTCGGTGATAACGGTCTTACGGATGATGATATTTACACATTACAGGGATATAATCAAGGCCACTACAGATAGTGGTCGTTATGGATGTCAT‑TGTCTATACGAGATGCTCCTACGTGAAATCTGAAAGTTAACGGGAGGCATTATGCTAGAATTTTTACGTAAGCTAATCCCTTGGGTTCTCGCTGGGATGCTATTCGGGGTAGGATGGCATCTAGGGTCAGACTCAATGGACGCT‑AATGGAAACAGGAGGT‑‑ACAATGAGTACGTTAagag                                                                                                                                          <  1:355759/276‑1 (MQ=255)`  
                                                                                                                                                                   `aaaacagCTCTTGAAGCGTACAAAGAGAAAGTCGGTGATAACGTTATTACGGATGATGATATTTACCCATTACAGTGATATACTAAAGGCCACTACAGCTAGTGGTCTTTATTGATGTCAT‑TTTCTATACGAGATGCTCCTACGTGACATCGGAAAGTTAACGGGAGGCATTATGCTAGAATTTTTACGTAAGCTAATCCCTTGGGTTCTCGCTGGGATGCTATTCGGGTTAGGATGGCATCTAGGGTAAGCCTCAATGGACGCTAAATGGAAACAGGAGGT‑‑ACAATGAGTACGTTAagag                                                                                                                                          <  1:31980/295‑1 (MQ=255)`  
                                                                                                                                                                    `cTGGTGCTCTTGAAGAGTACAAAGAGAAAGTCGGTGATAACGGTCTTACGGATGATGATATTTACACATTACAGTGATATACTCAAGGCCACTACAGATAGTGGTCTTTATGGATGTCAT‑TGTCTATACGAGATGCTCCTACGTGAAATCTGAAAGTTAACGGGAGGCATTATGCTAGAATTTTTACGTAAGCTAATCCCTTGGGTTCTCGCTGGGATGCTATTCGGGTTAGGATGGCATCTAGGGTCAGCCTCAATGGACGCTAAATGGAAACAGGAGGT‑‑ACAATGAGTACGTTAagaga                                                                                                                                         >  1:221625/1‑301 (MQ=255)`  
                                                                                                                                                                     `tGGTGTTTTTTAAGAGTAAAAATAGAAAGTCGGTGATAACGTTCTTACGTATGATGATATTTCACCATTAACGTGATTTACTCAATGCCACTACAGATATTGGTCTTTATGGATGTCAT‑TGTCTTTACGAGATGCTCCTACGTGAAATCTGAAAGGTAACGGGAGTCATTATGCTAGAATTTTTACGTAAGCTAATCCCTTGGGTTCTCGCTGGGATGCTATTCGGGTTAGGATGGCATCTAGGGTCAGACTCAATGGACGCTAAATGGAAACAGGAGGTACACAATGAGTACGTTAagag                                                                                                                                          <  1:151957/301‑1 (MQ=255)`  
                                                                                                                                                                     `cggagatgtgtataagagacaggagaAAGTTGGTGATAACGGTCTTACGGATGATGATATTTACACATTACAGTGATATACTCAAGGCCACTACAGATAGTGGTCTTTATGGATGTCAT‑TGTCTATACGAGATGCTCCTACGTGAAATCTGAAAGTTAACGGGAGGCATTATGCTAGAATTTTTACGTAAGCTAATCCCTTGGGTTCTCGCTGTGATGCTATTCGGGTTAGGATGGCATCTAGGGTCAGACTCAATGGACGCTAAATGGAAACAGGAGGTACACAATGAGTACGTTAagag                                                                                                                                          <  1:180802/279‑1 (MQ=255)`  
                                                                                                                                                                          `ctctTGAAGAGTACAAAGAGAAAGTCGGTGATAACGGTCTTACGGATGATGATATTTACACATTACAGTGATATACTCAAGTCCACTACAGATAGTGGTCTTTATGGATGTCAT‑TGTCTATACGAGATGCTCCTACGTGAAATCTGAAAGTTAACGGGAGGCATTATGCTAGAAGTTTTACGTAAGCTAATCCCTTGGGTTCTCGCTGGGATGCTATTCGGGTTAGGATGGCATCTAGGGTCAGACTCAATGGACGCTAAATGGAAACAGGAGGT‑‑ACAAGGAGTCCGTTAAGAGGGTTGAg                                                                                                                                   >  1:303218/1‑301 (MQ=255)`  
                                                                                                                                                                               `gAAGAGTACAAAGAGAAAGTCGGTGATAACGGTCTTACGGATGATGATATTTACACATTACAGTGATATACTCAAGGCCACTACAGATAGGGGTCTTTATGGATGTCAT‑TGTCTATACGAGATGCTCCTACGTGAAATCTGAAAGTTAACGGGAGGCATTATGCTAGAATTTTTACGTAAGCTAATCCCTTGGGTTCTCGCTGGGATGATATTCGTGTTAGGATGGAATCTAGGGTCAGACTGAATGGACGCGAAATGGAAACAGGTGGT‑‑ACAATAAGTACGTTAAGAGAGTGGAGGCTgg                                                                                                                              >  1:418742/1‑300 (MQ=255)`  
                                                                                                                                                                                    `gTACAAAGAGAAAGTCGGTGATAACGGTCTTACGGATGATGATATTTACACATTACAGTGATATACTCAAGGACACTACAGATAGTGGTCTTTATGGATGTCAT‑TGTCTATACGAGATGCTCCTACGTGAAATCTGAAAGTTAACGGGAGGCATTATGCTAGCATTTTTACGTAAGCTAATCCCTTGGGTTCTCGCTGGGATGCTATTCGGGTTAGGATGGCATCTAGGGTCAGACTCAATGGACGCTAAATGGAAACAGGAGGT‑‑ACAATGCGTACGTTAAGAGAGTTGAGGCTGCGAaga                                                                                                                         >  1:203253/1‑301 (MQ=255)`  
                                                                                                                                                                                                `aGTCGGTTATAACGGTCTTACTGATGATGATATTTACACATTACATTGATATACTCAAGGCCACTACAGATAGTGGTCTTTATGGATGTCAT‑TGTCTATACGAGATGCTCCTACGTGAAATCTGAAAGTTAACGGGAGGCATTATGCTAGAATTTTTACGTAAGCTAATCCCTAGGGTTCTCGCTGGGATGCTATTCGGGTTAGGATGGCATCTAGGGTCAGACTCAATGGACGCTAAATGGAAACAGGAGGT‑‑ACAATGAGTACGTTAAGAGAGTTGAGGCTGCGAAGAGCACTCAAagag                                                                                                             <  1:86350/301‑1 (MQ=255)`  
                                                                                                                                                                                                `aGTCGGTGATAACGGTCTTACGGATGATGATATTTACACATTACAGTGATATACTCAAGGCCACTACAGATAGTGGTCTTTATGGATGTCAT‑TGTCTATACGAGATGCTCCTACGTGAAATCTGAAAGTTAACGGGAGGCATTATGCTAGAATTTTTACGTAAGCTAATCCATTGGGTTCTCGCTGGGATGCTATTCGGGTTAGGATGGCATCTAGGGTCAGACTCAATGGACGCTAAATGGAAACAGGAGGT‑‑ACAATGAGTACGTTAAGAGAGTTGAGGCTGCGAAGAGCACTCAAagag                                                                                                             <  1:11678/301‑1 (MQ=255)`  
                                                                                                                                                                                                            `cGGTCTTACGGATGATGATATTTACACATTACAGTGATATACTCAAGGCCACTACAGATAGTGGTCTTTATGGATGTCAT‑TGTCTATACGAGATGCTCCTACGTGAAATCTGAAAGTTAACGGGAGGCATTATGCTAGAATTTTTACGTAAGCTAATCCCTTGGGTTCTCGCTGGGATGCTATTCGGGTTAGGATGGCATCTAGGGTCAGACTCAATGGACGCTAAATGGAAACAGGAGGT‑‑ACAATGAGTACGATAAGAGAGTTGAGGCTGCGAAGAGCACTCAAAGAGCAATCGATGCgg                                                                                                 >  1:14433/1‑301 (MQ=255)`  
                                                                                                                                                                                                            `cGGTCTTACGGATGATGATATTTACACATTACAGTGATATACTCAAGGCCACTACAGATAGTGGTCTTTATGGATGTCAT‑TGTCTATACGAGATGCTCCTACGTGAAATCTGAAAGTTAACGGGAGGCATTATGCTAGAATTTTTACGTAAGCTAAACACTTGGGTTCTCGATGGGATGCTATTCGGATTAGGATGGAATCTAGGGTCAGACTCAATGGACGCTAAATGGAAACAGGAGGTACACAATGAGTACGTTAATAGAGTTGAGGCTGCGAAGAGCACTCaactgtctcttataca                                                                                                   >  1:8119/1‑287 (MQ=255)`  
                                                                                                                                                                                                              `gTCTTACGGATGATGATATTTACACATTACAGTGATATACTCAAGGCCACTACAGATAGTGGTCTTTATGGATGTCAT‑TGTCTATACGAGATGCTCCTACGTGAAATCTGAAAGTTAACGGGAGGCATTATGCTAGAATTTTTACGTAAGCTAATCCCTTGGGTTCTCGCTGGGATGCTATTCGGGTTAGGATGGCATCTAGGGTCAGACTCAATGGACGCTAAATGGAAACAGGAGGT‑‑ACAATGAGTACGTTAAGAGAGTTGAGGCGGCGAAGAGCACTAAAAGAGCAATCGATGCGGTa                                                                                               >  1:104853/1‑301 (MQ=255)`  
                                                                                                                                                                                                               `tCTTACGGATGATGATATTTACAAATTACAGTGATATACTCAAGGCCACTACAGATAGTGGTCTTTATGGATGTCAT‑TGTCTATACGAGATGCTCCTACGTGAAAGCTGAAAGTTAACGGGAGGCATTATGCTAGAATTTTTACGTAAGCTAATCCCTTGGGTTCTCGCTGGGATGCTATTCGGGTTAGGATGGCATCTAGGGTCAGACTCAATGGACGCTAAATGGAAACAGGAGGT‑‑ACAATGAGTACGTTAAGAGAGTTGAGGCTGCGAAGAGCACTCAAAGAGCAATCGATGCGGTAt                                                                                              <  1:160998/301‑1 (MQ=255)`  
                                                                                                                                                                                                                    `cGGATGATGATATTTACACATTACAGTGATATACTCAAGGCCACTACAGATAGTGGTCTTTATGGATGTCAT‑TGTCTATACGCGATGCTCCTACGTGAAATCTGAAAGTTAACGGGAGGCATTATGCTAGAATTTTTACGTAAGCTAATCCCTTGGGTTCTCGCTGGGATGCTATTCGGGTTAGGATGGCATCTAGTGTCAGACTCAATGGACGCTAAATGGAAACAGGAGGT‑‑ACAATGAGTACGTTAAGAGAGTTGAGGCTGCGAAGAGCACTCAAAGAGCAATCGATGCGGTATCTGCt                                                                                         <  1:52899/301‑1 (MQ=255)`  
                                                                                                                                                                                                                      `gatgatgatATTTACACATTACAGTGATATACTCAAGGCCACTACAGATAGTGGTCTTTATGGATGTGAT‑TGTCTCTACGAGATGCTCCTACGTGAAATCTGAAAGTTAACGGGAGGCATTATGCTAGAATTTTTACGTAAGCTAATCCATTGGGTTCTCGCTAGGATGCTATTCCGGTTCGGATGGCATCTAGGGTCAGACTCAATGGCCGCTAAATGGCATCAGTAGGT‑‑ACAATGCGTACGTTAAGCGAGTTGAGGCTGAGAAGAGCACTCACAGAGCACTCGATGTGGTATTTgactg                                                                                       >  1:139519/1‑297 (MQ=255)`  
                                                                                                                                                                                                                        `tgatgatATTTACACATTACAGTGATATACTCAAGGCCACTACAGATAGTGGTCTTTATGGATGTCAT‑TGTCTATACGAGATGCTCCTACGTGAAATCTGAAAGTTAACGGGAGGCATTATGCTAGAATTTTTACGTAAGCTAATCCCTTTGGTTCTCGCTGGGATGCTATTCGGGTTAGGATGGCATCTAGGGTCAGACTCAATGGACGCTAAATGGAAACAGGAGGTACACAATGAGTACGTTAAGAGAGTTGAGGCTGCGAAGAGCACTCAAAGAGCAATCGATGCGGTATCTGCTaa                                                                                       <  1:442643/301‑1 (MQ=255)`  
                                                                                                                                                                                                                        `gtgtaaagagacagcaTTACAGTGATATACGCAAGGCCACTACAGATAGTGGTCTTTATGGATGTCAT‑TGTCTATACGAGATGCTCCTACGTGAAATCTGAAAGTTAACGGGAGGCATTATGCTAGAATTTTGACGTAAGCTAATCTCTTGGGTTCTCGCTGGGATGCTATTCGGGTTAGGATGGCATCTAGGGTCAGACTCAATGGACGCTAAATGGAAACAGGAGGT‑‑ACAATGAGTACGTTAAGAGAGTTGAGGCTGCGAAGAGCACTCAAAGAGCAATCGATGCGGTATCTGCTAAGt                                                                                     <  1:467433/287‑1 (MQ=255)`  
                                                                                                                                                                                                                            `tataagagacagttACAGTGATATACTCAAGGCCACTACAGATAGTGGTCTTTATGGATGTCAT‑TGTCTATACGAGATGCTCCTACGTGAAATCTGAAAGTTAACGGGAGGCATTATGCTAGAATTTTTACGTAAGCTAATCCCTTGGGTTCTCGCTGGGATGCTATTCGGGTTAGGATGGCATCTAGGGTCAGACTCAATGGACGCTAAATGGAAACAGGAGGTACACAATGAGTACGTTAAGAGAGTTGAGGCTGCGAAGAGCACTCAAAGAGCAATCGATGCGGTATCTGCTAAGTAt                                                                                   <  1:215954/289‑1 (MQ=255)`  
                                                                                                                                                                                                                              `tatTTACACATTACAGTGATATACTCAAGGCCACTACAGATAGTGGTCTTTTTGGATGTCAT‑TGTCTATACGAGATGCTCCTACGTGAAATCTGAAAGTTAACGGGAGGCATTATGCTAGAATTTTTACGTAAGCTAATCCCTTGGGTTCTCGCTGGGATGCTATTCGGATTAGGATGGCATCTAGGGTCAGACTCAATGGACGCTAAATGGAAACAGGAGGT‑‑ACAATGCGTACGTTAAGAGAGTTGAGGCTGCGAAGAGCACTCAAAGAGAAACCGCTGCGGTAGCTGCTAAGTATCaag                                                                               >  1:473261/1‑301 (MQ=255)`  
                                                                                                                                                                                                                              `tatTTACACATTACAGTGATATACTCAAGGCCACTACAGATAGTGGTCTTTATGGATGTCAT‑TGTCTATACGAGATGCTCCTACGTGAAATCTGAAAGTTAACGGGAGGCATTATGCTAGAATTTTTACGTAAGCTAATCCCTTGGGTTCTCGCTGGGATGCTATTCGGGTTAGGATTGCATCTAGGGTCAGACTCAATGGACGCTAAATGGAAACAGTAGGT‑‑ACAATGAGTACGTTAAGAGAGTTGAGGCTGCGAAGAGCACTCAAAGAGCAATCGATGCGGTATCTGCTAAGTATCaaa                                                                               >  1:167097/1‑300 (MQ=255)`  
                                                                                                                                                                                                                              `tatTTACACATTACAGTGATATACTCAAGGCCACTACAGATAGTGGTCTTTATGGATGTCAT‑TGTCTATACGAGATGCTCCTACGTGAAATCTGAAAGTTAACGGGAGGCATTATGCTAGAATTTTTACGTAAGCTAATCCCTTGGGTTCTCGCTGGGATGCTATTCGGGTTAGGATGGCATCTAGGGTCAGACTCAATGGACGCTAAATGGAAACAGGAGGTACACAATGAGTACGTTAAGAGAGTTGAGGCTGCGAAGAGCACTCAAAGAGCAATCGATGCGGTATCTGCTAAGTATCa                                                                                 >  1:395047/1‑301 (MQ=255)`  
                                                                                                                                                                                                                              `tatTTACACATTACAGTGATATACTCAAGGCCACTACAGATAGTGGTCTTTATGGATGTCAT‑TGTCTATACGAGATGCTCCTACGTGAAATCTGAAAGTTAACGGGAGGCATTATGCTAGAATTTTTACGTAAGCTAATCCCTTGGGTTCTCGCTGGGATGCTATTCGGGTTAGGATGGCATCTAGGGTCAGACTCAATGGACGCTAAATGGAAACAGGAGGT‑‑ACAATGAGTACGTTAAGAGAGTTGAGGCTGCGAAGAGCACTCAAAGAGCAATCGATGCGGTATCTGCTAAGTATAaag                                                                               >  1:164175/1‑301 (MQ=255)`  
                                                                                                                                                                                                                              `tatTTACACATTACAGTGATATACTCAAGGCCACTACAGATAGTGGTCTTTATGGATGTCAT‑TGTCTATACGAGATGCTCCTACGTGAAATCTGAAAGTTAACGGGAGGCATTATGCTAGAATTTTTACGTAAGCTAATCCCTTGGGTTCTCGCTGGGATGCTATTCGGGTTAGGATGGCATCTAGGGTCAGACTCAATGGACGCTAAATGGAAACAGGAGGT‑‑ACAATGAGTACGTTAAGAGAGTTGAGGCTGCGAAGAGCACTCAAAGAGCAATCGATGCGGTATCTGCTAAGTATAaag                                                                               >  1:398828/1‑301 (MQ=255)`  
                                                                                                                                                                                                                              `gtgtataagagacagttGATATACTCAAGGCCACTACAGATAGGTGTCTTTATGGATGTCAT‑TGTCTATACGAGATGCTCCTACGTGAAATCTGAAAGTTAACGGGAGGCATTATGCTAGAATTTTTACGTAAGCTAATCCCTTGGGTTCTCGCTGGGATGCTATTCGGGTTAGGATGGCATCTAGGTTCAGACTCAATGGACGCTAAATGGAAACAGGAGGT‑‑ACAATGAGTACGTTAAGAGAGTTGAGGCTGCGAAGAGCACTCAAAGAGCAATCGATGCGGTATCTGCTAAGTATCaag                                                                               <  1:156208/285‑1 (MQ=255)`  
                                                                                                                                                                                                                               `ttttACACAGTACAGTGATATACTCAAGGCCACTACAGATAGTGGTCTTTATGGATGTCAT‑TGCCTATACGAGATGCTCCAACGTGAAATCTGAAAGTTAACGGGAGGCATTATGCTAGAATTTTTACGTAAGCTAATCCCTTGGGTTCTCGCTGGGATGCTATTCGGGTTAGGATGGCATCTAGGTTCAGACTCAATGGACGCTAAATGGAAACAGGAGGT‑‑ACAATGAGTACGTTAAGAGAGTTGAGGCTGCGAAGAGCACTCAAAGAGCAATCGATGCGGTATCTGCTAAGTATCaaga                                                                              <  1:65030/300‑1 (MQ=255)`  
                                                                                                                                                                                                                               `tgtataagagacaggTGATATACTCAAGGCCACTACAGATAGTGGTCTTTATGGATGTCAT‑TGTCTATACGAGATGCTCCTACGTGAAATCTGAAAGTTAACGGGAGGCATTATGCTAGAATTTTTACGTAAGCTAATCCCTTGGGTTCTCGCTGGGATGCTATTCGGGTTAGGATGGCATCTAGGGTCAGACTCAATGGACGCTAAATGGAAACAGGAGGT‑‑ACAATGAGTACGTTAAGAGAGTTGAGGCTGCGAAGAGCACTCAAAGAGCAATCGATGCGGTATCTGCTAAGTATCaaga                                                                              <  1:350756/287‑1 (MQ=255)`  
                                                                                                                                                                                                                               `atTTACACATTACAGTGATATACTCAAGGCCACTACAGATAGTGGTCTTTATGGATGTCAT‑TGTCTATACGAGATGCTCCTACGTGAAATCTGAAAGTTAACGGGAGGCATTATGCTAGAATTTTTACGTAAGCTAATCCCTTGGGTTCTCGCTGGGATGCTATTCGGGTTAGGATGGCATCTAGGGTCAGACTCAATGGACGCTAAATGGAAACAGGAGGT‑‑ACAATGAGTACGTTAAGAGAGTTGAGGCTGCGAAGAGCACTCAAAGAGCAATCGATGCGGTATCTGCTAAGTATCaaga                                                                              <  1:477963/301‑1 (MQ=255)`  
                                                                                                                                                                                                                                `ttataaggagcaggTGAAATACTCAAGGCCACTACAGATAGGGGGCGTTATGGAGGTCAT‑TGTCTATACGAGATGCGCCTACGTGAAATCTGAAAGTTAACGGGAGGCATTATGCTAGAATTTTTACGTAAGCTAATCCCTTGGGTTCTCGCTGGGATGCTATTCGGGTTAGGATGGCATCGAGGGTCAGACTCAATGGACGCTAAATGGAAACAGGAGGT‑‑ACAATGAGTACGTTAAGAGAGTTGAGGCTGCGAAGAGCACTCAAAGAGCAATCGATGCGGTATCTGCTAAGTATCaagaa                                                                             <  1:345160/288‑1 (MQ=255)`  
                                                                                                                                                                                                                                   `aaaaagtagtgtgatgtcagcAAGGCCGCTACAGATAGTGGTCTTTATGGATGTCAT‑TGTCTAGACGTAATGATAGTACGTGAAATCTGAAAGTTAACGGGAGGCATTATGCTAGAAGTTTGACGTAAGCTAATCCCTGGGGTTCTTGATGGGATGCTATTCGGGTTAGGATGGCATCTAGGGTCAGACTCAATGGACGATAAATGGAAACAGGAGG‑‑GACAATGAGTACGATAAGAGAGTTGAGGCTGCGAAGAGCACTCAAAGAGCAATCGATGCGGTATCTGCTAAGTATCAAGAAGAc                                                                          <  1:427147/281‑1 (MQ=255)`  
                                                                                                                                                                                                                                               `gATATACTCAAGGCCACTACAGATAGTGGTCTTTATGGATGTCAT‑TGTCTATACGAGATTCTCCTACGTGAAATCTGAAAGTTAACGGGAGGCATTATGCTAGAATTTTTACGTAAGCTAATCCCTTGGGTTCTCGCTGGGATGCTATTCGGGTTAGGATGGCATCTAGGGTCAGACTCAATGGACGCTAAATGGAAACAGGAGGT‑‑ACAATGAGTACGTTAAGAGAGTTGAGGCTGCGAAGAGAACTCAAAGAGCAATCGATGCGGTATCTGCTacgctgtctcttatacacatctcctag                                                              >  1:457290/1‑275 (MQ=255)`  
                                                                                                                                                                                                                                               `gATATACTCAAGGCCACTACAGATAGTGGTCTTTATGGATGTCAT‑TGTCTATACGAGATGCTCCTACGTGAAATCTGAAAGTTAACGGGAGGCATTATGCTAGAATTTTTACGTAAGCTAATCCCTTTGGTTCTCGCTGGGATGCTATTCGGGTTAGGATGGCATCTAGGGTCAGACTCAATGGCCGCTAAATGGCAACAGGAGGT‑‑ACAATGAGTACGTTAAGAGAGTTGAGGCTGCGAAGAGCACTCAAATAGCAATCGATGCGGTATCTGCTAAGTATCaagcctgtctcttatacaca                                                              >  1:434862/1‑284 (MQ=255)`  
                                                                                                                                                                                                                                                  `gtgtataagagacagaccgATAGTGGTCTTTATGGATGTCAT‑TGTCTATACGAGATGCTCCTACGTGAAACCTGAAAGTTAACGGGAGGCATTATGCTAGAATTTTTACGTAAGCTAATCGCTTGGGTTCTCGCTGGGATGCTATTAGGGTTAGGATGGCATCTAGAGTCAGACTAAATGGACGCTAAATAGAAACAGGAAGT‑‑ACAATGAGTACGTTAAGAGAGTTGAGGCTGCGAAGAGCACTCAAAGAGCAATCGATGCGGTATCTGCTAAGTATCAAGAAGACCTTGCAGCGCTGGaa                                                           <  1:207576/283‑1 (MQ=255)`  
                                                                                                                                                                                                                                                   `taCTCAAGGCCACTACAGATAGTGGTCTTTATGGAGTTCAT‑TGTCTATACGAGATGCTCCTACGTGAAATCTGAAAGTTAACGGGAGGCATTATGCTAGAATTTTTACGTAAGCTAATCCCTTGGGTTCTCGCTGGGATGCTATTCGGGTTAGGATGGCATCTAGGGTCAGACTCAATGGACGCTAAATGGAAACAGGAGGT‑‑ACAATGAGTACGTTAAGAGAGTTGAGGCTGCGAAGAGCACTCAAAGAGCAATCGATGCGGTATCTGCTAAGTATCAAGAAGACCTTGCCGCGCTGGAAg                                                          <  1:483201/301‑1 (MQ=255)`  
                                                                                                                                                                                                                                                       `aaaGGCCATTGGAGATAGGTGACTTTATTGATGTCAT‑TGGATAAACGAGGTGATTCTACGTGAAATCTGAAAGTTAACGGGAGGCATTATGATAGAAATTTTACGTAAGCTAATCACTTGGGTTCTCGCTGGGCTTCTATTCGGGTTAGGATGGCATCGAGGCTCATGCTCAATGGACGCTAAATGGAAACAGGAGGTACACAATGATTACGTTAAGAGAGTTGATGCTGCGAAGAGCACTCAAAGAGCAATCGATGCTGTATCTGCTAAGTATCAAGAAGACCTTGCCGCGCTGGAAggg                                                        <  1:290114/300‑1 (MQ=255)`  
                                                                                                                                                                                                                                                            `ccACTACAGATAGTGGTCTTTATGGATGTCAT‑TGTCTATACGAGATGCTCCTACGTGAAATCTGAAAGTTAACTGGAGGCATTATGCTAGAATTTTTACGTAAGCTAATCCCTTGGGTTCTCGCTGGGATGCTATTCGGGTTAGGATGGCATCTAGGGTCAGACTCAATGGACGCTAAATGGAAACAGGAGGT‑‑ACAATGAGTACGTTAAGAGAGTTGAGGCTGCGAAGAGCACTCAAAGAGCAATCGATGCGGTATCTGCTAAGTATCAAGAAGACCTTGCCGCGCTGGAAGGGAGCACTg                                                 >  1:379006/1‑301 (MQ=255)`  
                                                                                                                                                                                                                                                            `atgtgtataagagacagcTTTATGGATGTCAT‑TGTCTATACGAGATGCTCCTACGTGAAATCTGAAAGTTAAAGGGAGGCATTATGCTAGAATTTTTACGTAAGCTAATCCCTTGGGTTCTCGCTGGGATGCTATTCGGGTTAGGATGGCATCTAGGGTCAGACTCAATGGACGCTAAATGGAAACAGGAGGT‑‑ACAATGAGTACGTTAAGAGAGTTGAGGCTGCGAAGAGCACTCAAAGAGCAATCGATGCGGTATCTGCTAAGTATCAAGAAGACCTTGCCGCGCTGGAAGGGAGCACTg                                                 <  1:365942/284‑1 (MQ=255)`  
                                                                                                                                                                                                                                                              `aCTATAGATAGTGGTCTTTATGGATGTCAT‑TGTCTATACGAGATGCTCCTACGTGAAATCTGAAAGTTAACGGGAGGCATTATGCTAGAATTTTTACGTAAGCTAATCCCTTGGGTTCTCGCTGGGATGCTATTCGGGTTAGGATGGCATCTAGGGTCAGACTCAATGGACGCTAAATGGAAACAGGAGGTACACAATGAGTACGTTAAGAGAGTTGAGGCTGCGAAGAGCACTCAAAGAGCAATCGATGCGGTATCTGCTAAGTATCAAGAAGACCTTGCCGCGCTGGAAGGGAGCACTg                                                 >  1:130288/1‑301 (MQ=255)`  
                                                                                                                                                                                                                                                              `aCTACAGATAGTGGTCTTTATGGATGTCAT‑TGTCTATACGAGATGCTCCTACGTGAAATCTGAAATTTAACGGGAGGCATTATGCTAGAATTTTTACGTAAGCTAATCCCTTGTTTTCTCGCTGGGATGCTATTCGGGTTAGGATGTCATCTAGGGTCAGTCTCAATGGACGCTAAATGGAAACAGGAGTT‑‑ACAATGATTACTTTAAGAGATTTGAGGCTGCGAAGATCACTCAAAGAGCAATCGATGCGGTATCTGCTAAGTATCAAGAAGACCTTGCCGCGCTGGAAGGGCGCAcctgt                                               >  1:285757/1‑297 (MQ=255)`  
                                                                                                                                                                                                                                                               `cTACAGATAGTGGTCTTTATGGATGTCAT‑TGTCTATACGAGATGCTCCTACGTGAAATCTGAAAGTTAACGGGAGGCATTATGCTAGAATTTTTACGTAAGCTAATCCCTTGGGTTCTCGCTGGGATGCTATTCGGGTTATGATGGCATCTAGGGTCAGACTCAATGGACGCTAAATGGAAACAGGAGGTACACAATGAGTACGTTAAGAGAGTTGAGGCTGCGAAGAGCACTCAAAGAGCAATCGATGCGGTATCTGCTAAGTATCaagactgtctcttatacacatctccgagccgacg                                                >  1:293734/1‑271 (MQ=255)`  
                                                                                                                                                                                                                                                               `cTACAGATAGTGGTCTTTATGGATGTCAT‑TGTCTATACGAGATGCTCCTACGTGAAATCTGAAAGTTAACGGGAGGCATTATGCTAGAATTTTTACGTAAGCTAATCCCTTGGGTTCTCGCTGGGATGCTATTCGGGTTAGGATGGCATCTAGGGTCAGACTCAATGGACGCTAAATGGAAACAGGAGGT‑‑ACAATGAGTACGTTAAGAGAGTTGAGGCTGCGAAGAGCACTCAAATAGCAATCGATGCGGTATCTGCTAAGTATCAAGAAGACCTTGCAGCGCTGGAAGGGAGCACTGATa                                              >  1:307802/1‑301 (MQ=255)`  
                                                                                                                                                                                                                                                               `cTACAGATAGTGGTCTTTATGGATGTCAT‑TGTCTATACGAGATGCTCCTACGTGAAATCTGAAAGTTAACGGGAGGCATTATGCTAGAATTTTTACGTAAGCTAATCCCTTGGGTTCTCGCTGGGATGCTATTCGGGTTAGGATGGCATCTAGGGTCAGACTCAATGGACGCTAAATGGAAACAGGAGGT‑‑ACAATGAGTACGTTAAGAGAGTTGAGGCTGCGAAGAGCACTCAAAGAGCAATCGATGCGGTATCTGCTAAGTATCAAGCAGACCCTgtctcttatacacatctccgagcac                                              >  1:297985/1‑277 (MQ=255)`  
                                                                                                                                                                                                                                                               `cTACAGATAGTGGTCTTTATGGATGTCAT‑TGTCTATACGAGATGCTCCTACGTGAAATCTGAAAGTTAACGGGAGGCATTATGCTAGAATTTTTACGTAAGCTAATCCCTTGGGTTCTCGCTGGGATGCTATTCGGGTTAGGATGGCATCTAGGGTCAGACTCAATGGACGCTAAATGGAAACAGGAGGT‑‑ACAATGAGTACGTTAAGAGAGTTGAGGAGGGGAAGAGCACTCAAAGAGCAATCGATGCGGAGTCTGCGAAGTATAAAGAAGACCtgtatcgaatacaaatctccgagccca                                              >  1:488095/1‑275 (MQ=255)`  
                                                                                                                                                                                                                                                                `taagagacagggTCTTGATGGATGTAAT‑TGTCTATACGAGATGATCCTACGTGAAATCTGAAAGTTAACGGGAGGCATTAGGCTAGAATGTTTACGTAAGCTAATCCCTTGGGTTCTCGCTGGGATGCTATTCGGGTTAGGATGGCATCTAGGGTCAGACTCAATGGACGCTAAATGGAAACAGGAGGT‑‑ACAATGAGTACGTTAAGAGAGTTGAGGCTGCGAAGAGCACTCAAAGAGCAATCGATGCGGTATCTGCTAAGTATCAAGAAGACCTTGCCGCGCTGGAAGGGAGCACTGATAg                                             <  1:340371/291‑1 (MQ=255)`  
                                                                                                                                                                                                                                                                `tACAGATAGTGTTCTTTATGGATGTCAT‑TGTCTATACGAGATGCTCCTACGTGAAATCTGAAAGTTAACGGGAGGCATTATGCTAGAATTTTTACGTAAGCTAATCCCTTGGGTTCTCGCTGGGATGCTATTCGGGTTAGGATGGCATCTAGGGTCAGACTCAATGGACGCTAAATGGAAACAGGAGGT‑‑ACAATGAGTACGTTAAGAGAGTTGAGGCTGCGAAGAGCACTCAAAGAGCAATCGATGCGGTATCTGCTAAGTATCAAGAAGACCTTGCCGCGCTGGAAGGGAGCACTGATAg                                             <  1:229665/301‑1 (MQ=255)`  
                                                                                                                                                                                                                                                                `gtgtataagagacagttAGGGATGTCAT‑TGTCTATACGAGATGCTCCTACGTGAAATCTGAAAGTTAACGGGAGGAATTATGCTAGAATTTTTACGTAAGCTAATCCCTGGGGTTCTCGCTGGGATGCTATTCGGGTTAGGATGGCATCTAGGGTCAGACTCAATGGACGCTAAATGGAAACAGGAGGT‑‑ACAATGAGTACGTTAAGAGAGTTGAGGCTGCGAAGAGCACTCAAAGAGCAATCGATGCGGTATCTGCTAAGTATCAAGAAGACCTTGCCGCGCTGGAAGGGAGCACTGATAg                                             <  1:135146/286‑1 (MQ=255)`  
                                                                                                                                                                                                                                                                   `aGATAGTGGTCTTTATGGATGTCAT‑TGTCTATACGAGATGCTCCTACGTGAAATCTGAAAGTTAACGGGAGGCATTATGCTAGAATTTTTACGTAAGCTAATCCCTTGGGTTCTCGCTGGGATGCTATTCGGGTTAGGATGGCATCTAGGGTCAGACTCTATGGACGCTAAATGGAAACAGGAGGT‑‑ACAATGAGTACGTTAAGAGAGTTGAGGCTGCGAAGAGCACTCAAAGAGCAATCGATGCGGTATCTGCTAAGTATCAAGAAGACCTTGCCGCGCTGGAAGGGAGCACTGATactgt                                          >  1:328059/1‑297 (MQ=255)`  
                                                                                                                                                                                                                                                                        `gTGGTCTTTATGGATGTCAT‑TGTCTATACGAGATGCCCCTACGTGAAATCTGAAAGTTAACGGGAGGCATTATGCTAGAATTTTTACGTAAGCTAATCCCTTGGGTTCTCGCTGGGATGCTATTCGGGTTAGGATGGCATCTAGGGTCAGACTCAATGGACGCTAAATGGAAACAGGAGGTACACAATGAGTACGCTAAGAGAGTTGAGGCTGCGCAGAGCACTCAAAGAGCAATCGATGCGGTATCTGCTAAGTATCAAGAAGACCTTGCCGCGCTGGAAGGGTGCACTGATAGGattat                                       >  1:336113/1‑301 (MQ=255)`  
                                                                                                                                                                                                                                                                          `gtcgcagatgtgtataagatacagataCGAGATGCTACTACGTGAAATCTGAAAGTTAACGGTAGGAATTATGCTAGAATTTTTACGTAAGCTAATCCCTTGGGTTCTCGCTGGGATGCTATTCGTGTTAGGATGGCATCTAGGGTCAGACTCAATGGACGCTAAATGGAAACAGGATGT‑‑ACAATGAGTACGTTAAGAGAGTTGAGGCTGCGAAGAGCACTCAAAGAGCAATCGATGCGGTATCTGCTAAGTATCAAGAAGACCTTGCCGCGCTGGAAGGGAGCACTGATAGGATTATTTc                                    <  1:492616/277‑1 (MQ=255)`  
                                                                                                                                                                                                                                                                              `tttATGCATGTCCTGTGT‑TATACGAGATGCTTCTACGTGAAATCTGACAGTTAACCGGAGGCATTATGCTAGCATTTTTACGTAATCTAATCCCTTGGCTTCTCGCTGAGATGCTATTCTGGTTAGGATTGCATCGAGGGTCATACTCAATGGACGCTAAATGGAAACAGGAGGT‑‑ACAATGCGTACGTTAAGAGAGTTGAGGCGGCGAAGAGCACTCAAAGAGCAATCGATGCCGTATCTGCTAAGTATCAAGGAGACCTGGCCGCGCTGGAAGGTAGCACTGATAGGATTATTTCTGAtt                               <  1:17191/301‑1 (MQ=255)`  
                                                                                                                                                                                                                                                                                `tATGGCTGTCAT‑TGTCTATACGAGATGCTCCTACGTTAAATCTGAAAGTTAACGTGAGGCATTATGCTAGGATTTTTACGTAAGCTAATCCCTTGGGTTCTCGCTGGGATGCTATTCGGGTTAGGATGGCATCTAGGGTCAGACTCAATGGACGCTAAATGGAAACAGGAGGTACACAATGAGTACGTTAAGAGAGTTGATGCTGCGAAGAGCACTCAAAGAGCAATCGATGCGGTATCTGCTAAGTATCAAGAAGACCTTGCCGCGCTGGAAGGGAGCACTGATAGGATTATTTCTGAtt                               <  1:261661/301‑1 (MQ=255)`  
                                                                                                                                                                                                                                                                                   `ggATGTCAT‑TGTCTATACGAGATGCTCCTACGTGAAATCTGAAAGTTAACGGGAGGCATTATGCTAGAATTTTTACGTAAGCTAATCCCTTGGGTTCTCGCTGGGATGCTATTCGGGTTAGGATGGCATCTAGGGTCAGACTCAATGGACGCTAAATGGAAACAGGAGGT‑‑ACAATGGGTACGTTAAGAGAGTTGAGGATGCGAAGAGCACTAAAAGAGCAATCGATGCGGTATCTGCTCAGTATCAAGAAGACCTTGCAGCGCTGGAAGGGAGCACTGATAGGATTATTACTGATTTGCGt                          >  1:61239/1‑301 (MQ=255)`  
                                                                                                                                                                                                                                                                                   `ggATGTCAT‑TGTCTATACGAGATGCTCCTACGTGAAATCTGAAAGTTAACGGGAGGCATTATGCTAGAATTTTTACGTAAGCTAATCCCTTGGGTTCTCGCTGGGATGCTATTCGGGTTAGGATGGCATCTAGGGTCAGACTCAATGGACGCTAAATGGAAACAGGAGGT‑‑ACAATGAGTACGTTAAGAGAGTTGAGGCTGCGAAGAGCACTCAAAGAGCAATCGATGCGGTATCTGCTAAGTATCAAGAAGACCTTGCCGCGCTGGAAGGGAGCACTGATAGGATTATTTCTGATTTGCGt                          <  1:190313/301‑1 (MQ=255)`  
                                                                                                                                                                                                                                                                                     `aTGTCAT‑TGTCTATACGAGATGCTCCTACGTGAAATCTGAAAGTTAACGGGAGGCATTATGCTAGAATTTTTACGTAAGCTAATCCCTTGGGTTCTCGCTGGGATGCTATTCGGGTTAGGATGGCATCTAGGGTCAGACTCAATTGACGCTAAATGGAAACAGGAGGTACACAATGAGTACGTTAAGAGAGTTGAGGCTGCGAAGAGCACTCAAAGAGCAATCGATGCGGTATCTGCTAAGTATCAAGAAGACCTTGCCGCGCTGGAAGGGAGCACTGATAGGATTATTTCTGATTTGCGt                          <  1:24311/301‑1 (MQ=255)`  
                                                                                                                                                                                                                                                                                      `atggaattgtattaaCGGGATGCGCCTACGTGAAATCTGAAAGTTCACGGGGGGCATTATGCTAGAATTTTTACGTAAGCTAATCCCTTGTGTTATGGCTGGGATGCTATTCGGGTTAGGATGGAGTCTAGGGTCAGACTCAATGGACGCTAAATGGAAACAGGAGGT‑‑ACAATGAGTACGTTAAGAGAGTTGAGGCTGCGAAGAGCACTCAAAGAGCAATCGATGCGGTATCTGCTAAGTATCAAGAAGACCTTGCCGCGCTGGAAGGGAGCACTGATAGGATTATTTCTGATTTGCGTAg                        <  1:281713/287‑1 (MQ=255)`  
                                                                                                                                                                                                                                                                                             `tGTCTATACGAGATGCTCCTACGTGAAATCTGAAAGTTAACGGGAGGCATTATGCTAGAATTTTTACGTAAGCTAATCCCTTGGGTTCTCGCTGGGATGCTATTCGGGTTAGGATGGCATCTAGGGTCAGACTCAATTGACGCTAAATGGAAACAGGAGGT‑‑ACAATGAGTACGTTAAGAGAGTTGAGGCTGCGAAGAGCACTCAAAGAGCAATCGATGCGGTATCTGCTAAGTATCAAGAAGACCTTGCCGCGCTGGAATGGAGCACTGATAggctgtctcttctacacctctccgagccc                 >  1:273276/1‑274 (MQ=255)`  
                                                                                                                                                                                                                                                                                              `gTCTATACGAGATTCTCCTACGTGAAATCTGAAAGTTAACGGGAGGCATTATGCTAGAATTTTTACGTAAGCTAATCCCTTGGGTTCTCGCTGGTATGCTATTCGGTTTAGGATGGCATCTAGGGTCAGACTCAATGTACGCTAAATGGAAACAGTAGGT‑‑ACAATGAGTACGTTCAGAGAGTTTAGGCTGCGAAGAGAACTCAAAGAGCAATCGATGCGGTATCTGCTAAGTATCAAGAATACCTTGCCCCGCTGGACAGGCTCAATGATAGGATTATTTCTGATTTGCTTAGCGACAATa                >  1:498685/1‑301 (MQ=255)`  
                                                                                                                                                                                                                                                                                              `gTCTATACGAGATGCTCCTACGTGAAATCTGAAAGTTAACGGGAGGCATTATGCTAGAATTTTTACGTAAGCTAATCCCTTGGGTTCTCGCTGGGATGCTATTCGGGTTAGGATGGCATCTAGGGTCAGACTCAATGGACGCTAAATGGAAACAGGAGGT‑‑ACAATGAGTACGTTAAGAGAGTTGAGGATGCGAAGAGCACTCAAAGAGCAATCGATGCGGTATCTGCTAAGTATCAAGAAGACCTTGCCGCGCTGGAAGGGAGCACTGATAGGATTATTTCTGATTTGCGTAGCGACAGTa                >  1:311845/1‑301 (MQ=255)`  
                                                                                                                                                                                                                                                                                              `agcTATACGAGATGCTCCTACGTGAAATCTGAAAGTTAACGGGAGGCATTATGCTAGAATTTTTACGTAAGCTAATCCCTTGGGTTCTCGCTGGGATGCTATTCGGGTTAGGATGGCATCTAGGGTCAGACTCAATGGACGCTAAATGGAAACAGGAGGT‑‑ACAATGAGTACGTTAAGAGAGTTGAGGCTGCGAAGAGCACTAAAAGAGCAATCGATGCGGTATCTGCTAAGTATCAAGAAGACCTTGCCGCGCTGGAAGGGAGCACTGATAGGATTATTTCTGATTTGCGTAGCGACAATa                <  1:390789/299‑1 (MQ=255)`  
                                                                                                                                                                                                                                                                                                  `ataCGAGGTGCTCCTACGTGAAATTTGAAAGTTAACGGGAGGCATTATGCTAGAATTTTTACGTAAGCTAATCCCTTGGGTTCTCGCTGGGATGCTATTCGGGTTAGGATGGCATCTAGGGTCAGACTCAATGGACGCTAAATGGAAACAGGAGGTACACAATGAGTACGTTAAGAGAGTTGAGGCTGCGAAGAGCACTCAAAGAGCAATCGATGCGGTATCTGCTAAGTATCAAGAAGACCTTGCCGCGCTGGAAGGGAGCACTGATAGGATTATTTCTGATTTGCGTAGCGACAATAAg              <  1:277269/301‑1 (MQ=255)`  
                                                                                                                                                                                                                                                                                                  `ataCGAGACGCTCCTATGTGAAATATGAAAGTTAACGGGAGGCATTATGCTAGAATTTTTACGTAAGCCAATCCCTGGGGTTCTCGCTGGGATGCTATTCGGGTTAGGATGGCATCTAGGGTCAGACTCAATGGACGCTAAATGGAAACAGGAGGT‑‑ACAATGAGTACGTTAAGAGAGTTGAGGCTGCGAATAGCACTCAAAGAGCAATCGATGCGGTATCTGCTAAGTATCAAGAAGACCTTGCCGCGCTTGAAGGGAGCACTGATAGGATTATTTCTGATTTGCGTAGCGACAATAAGCg            <  1:125525/301‑1 (MQ=255)`  

                                                                                                                                                                                                                                                                                                             `|`                                                                                                                                                                                                                                                                                                               
`CTTAGAGATGTTGGACACTGCGATGGCTCAGCGTATGCTTGCGGACCTTTCGGACCATGAGCGTCGCTCTCCGCAACTCTATAATGCTATTAACAAACTGTTAGACCGCCACAAGTTCCAGATTGGTAAGTTGCAGCCGGATGTTCACATCTTAGGTGGCCTTGCTGGTGCTCTTGAAGAGTACAAAGAGAAAGTCGGTGATAACGGTCTTACGGATGATGATATTTACACATTACAGTGATATACTCAAGGCCACTACAGATAGTGGTCTTTATGGATGTCAT‑TGTCTATACGAGATGCTCCTACGTGAAATCTGAAAGTTAACGGGAGGCATTATGCTAGAATTTTTACGTAAGCTAATCCCTTGGGTTCTCGCTGGGATGCTATTCGGGTTAGGATGGCATCTAGGGTCAGACTCAATGGACGCTAAATGGAAACAGGAGGTACACAATGAGTACGTTAAGAGAGTTGAGGCTGCGAAGAGCACTCAAAGAGCAATCGATGCGGTATCTGCTAAGTATCAAGAAGACCTTGCCGCGCTGGAAGGGAGCACTGATAGGATTATTTCTGATTTGCGTAGCGACAATAAGCGGTTGCGCGTC  >  NC_001604.1/36582‑37183`

  

Alignment Legend

Aligned base mismatch/match (shaded by quality score): `ATCG/ATCG` `< 3 ≤` `ATCG/ATCG` `< 10 ≤` `ATCG/ATCG` `< 18 ≤` `ATCG/ATCG` `< 32 ≤` `ATCG/ATCG` `< 38 ≤` `ATCG/ATCG`

Unaligned base: `atcg`    Masked matching base: `atcg`    Alignment gap: `‑`    Deleted base: `‑`

Reads not counted as support for junction

read\_name Not counted due to insufficient overlap past the breakpoint.

read\_name Not counted due to not crossing MOB target site duplication.
